# Three-dimensional directional nerve guide conduits fabricated by dopamine-functionalized conductive carbon nanofibre-based nanocomposite ink printing†

**DOI:** 10.1039/d0ra06556k

**Published:** 2020-11-09

**Authors:** Shadi Houshyar, Mamatha M. Pillai, Tanushree Saha, G. Sathish-Kumar, Chaitali Dekiwadia, Satya Ranjan Sarker, R. Sivasubramanian, Robert A. Shanks, Amitava Bhattacharyya

**Affiliations:** School of Engineering, College of Science, Engineering and Health, RMIT University Melbourne 3001 Australia shadi.houshyar@rmit.edu.au; Tissue Engineering Laboratory, PSG Institute of Advanced Studies Coimbatore-641004 India; Functional, Innovative and Smart Textiles, PSG Institute of Advanced Studies Coimbatore-641004 India abh@psgias.ac.in; RMIT Microscopy and Microanalysis Facility, College of Science, Engineering and Health, RMIT University Melbourne 3001 Australia; Department of Biotechnology and Genetic Engineering, Jahangirnagar University Savar Dhaka-1342 Bangladesh; Electrochemistry Laboratory, PSG Institute of Advanced Studies Coimbatore- 641004 India; School of Science, College of Science, Engineering and Health, RMIT University Melbourne 3000 Australia

## Abstract

A potential issue in current nerve guides is that they do not transmit electrical nerve impulses between the distal and proximal end of an injured nerve, *i.e.* a synapse. Conductivity is a desirable property of an ideal nerve guide that is being considered for peripheral nerve regeneration. Most conductive polymers reported for the fabrication of tissue engineering scaffolds, such as polypyrrole and polyaniline, are non-biodegradable and possess weak mechanical properties, and thus cannot be fabricated into 3D structures. Herein, we have designed a new nanocomposite material composed of dopamine, carbon nanofibers (CNF) and polycaprolactone (PCL) for the fabrication of nerve conduits, which facilitates the growth and migration of neurons toward the targeted end of an injured nerve. This support and navigation of the scaffold leads to better sensory and motor function. The results showed that the mechanical properties of the printed PCL increased by 30% in comparison with the pure PCL film, which is comparable with human nerves. The *in vitro* cell study of human glioma cells showed that the printed lines provided support for neural cell attachment, migration and differentiation toward the targeted end. In contrast, in the absence of printed lines in the scaffold, the cells attach and grow in random directions, forming a flower shape (cell cluster) on the surface of PCL. Thus, the proposed scaffold is a promising candidate for nerve guide application based on its signal transmission and navigating neurons in a correct pathway towards the targeted end.

## Abstract 2

Directional growth induced by dopamine-functionalized CNF-based nanocomposite ink printing.

## Graphical abstract

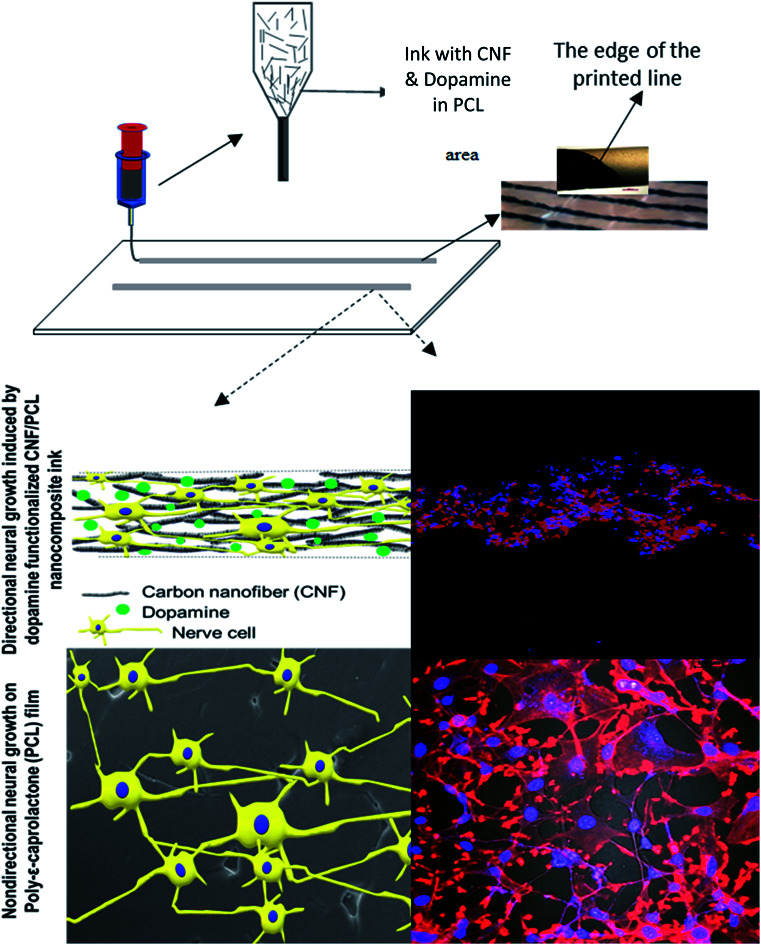

## 1. Introduction

The nervous system is divided into the brain, spinal cord (central nervous system, CNS) and nerve cells, which control voluntary and involuntary movements (peripheral nervous system, PNS). Nerve injury caused by trauma or physical insult is a common form of nerve lesion, and millions of cases are recorded worldwide annually.^1^ This type of injury can range from mild to severe and depends on the mode, severity and anatomical location of the impact and injury. It can lead to painful neuropathies, permanent disability, or significant disruption, which can result in failure of the sensory and motor functions in the body.^1–7^ The recovery of nerves include the migration and generation of new-born cells at the injured area; however, directing the growth and branching of axons to reform nerves is essential, together with the formation of functional synapses between the distal and proximal ends.^8^ After an injury to the nervous system, a degeneration process known as ‘Wallerian’ degeneration occurs. This means the newly regenerated axons, which are supported by local and produced cytoskeletal protein, grow toward the distal stump.^1,9^ Schwann cells align to direct the in-growing axons toward the distal stump, and this should occur within a favourable time to avoid Schwann cell denervation, which leads to slower axon growth.^10^ Therefore, for nerve injury with a large gap, an autologous graft or allograft should be used as a physical guide for directing axonal sprout, provide nutrients, and reduce scar tissue formations, while preventing excessive stretching or reduction of blood flow to the injured nerve.^1,11–14^ This treatment requires the harvesting of secondary nerves in the body prior to surgery, which can lead to the formation of painful neuromas, the structurally incompatibility of nerves, misalignment and mismatch of axonal growth, and even secondary site morbidity.^1^ Consequently, researchers and clinicians have focused their attention on alternative techniques, such as artificial nerve guides made of synthetic or natural polymers, to optimize recovery.^11,13,14^ However, alternative techniques such as nerve guides often fail due to the impossibility of duplicating the structure and bifunctionality of nerve tissue, resulting in inappropriate functional and sensory recovery, which is the main function of the nervous system.^1,14,15^ Thus, to overcome these issues for an effective nerve repair, Schwann cells and axonal growth should be directed to the targeted distal end. However, little is known about how these cells migrate, including how they can be directed to the distal end of damaged nerves after nerve guide implantation.^6,7,16^

Another way to overcome the above-mentioned issues is to improve nerve guides through the design of the methods and materials employed for their fabrication. This can be done *via* the addition of biological factors including growth and neurotrophic factors, extracellular matrix (ECM) components, myelin debris, and macrophage-derived cytokines, which improve axonal regeneration, Schwann cell proliferation with cell maintenance and differentiation, and sensory neuron survival until maturation.^6,7,14^ Dopamine (DA) is a neurotrophic factor (NTTF) that acts on DA receptors, and several studies have supported its potential for enhancing the survival, growth, maintenance, and differentiation of neurons.^4,6,7,10,12,14^ As reported by Kesteren *et al.*, dopamine is vital in axon growth and synapse formation during the embryonic stage and after injury.^17^ For example, Liu *et al.* demonstrated the potential enhancement of DA neurotrophic factors on peripheral nerve regeneration and morphology, and functional recovery in a rat sciatic nerve.^15^ This regeneration of neurons by DA was studied by Berg *et al.*, where the most extensive regenerative capacities among vertebrates were shown.^18^ They showed that DA neurons were formed within the first four weeks after ablation *via* gradual regeneration.^18^ Consistent with this, Parish *et al.* created a Parkinson's-like model, where complete regeneration of the dopaminergic cells occurred within 30 days, accompanied by the recovery of locomotor activity.^19^ However, the study by Lan *et al.* revealed the negative effect of a high concentration of dopamine on human glioma cells due to the inducement of apoptosis by the activation of the cytochrome c and caspase-dependent apoptotic pathways.^20^

It is well-known that a wide range of active and passive biological functions in the body are affected by the electrical signals generated by the nervous system. These signals are responsible for a range of functions, ranging from movement and thinking to sensory perception and respiration.^21,22^ These generated electrical signals named “bioelectricity” play an important role in maintaining the biological functions of the body, such as thinking, movement and sensing, and they can accelerate wound healing processes in the body. Therefore, the application of conductive materials, allowing free movement of electrons between atoms, to connect injured neural tissue after injuries, proximal and distal, will be beneficial.^1,4,23^ Several studies have demonstrated the positive effect of electro-conductive materials on the differentiation and regeneration of stem cells, including the extension of neurites in neural regeneration due to their ability to transfer bioelectrical signals between neurons and stimulate nerves.^3,18^ Gopinathan *et al.* reported the effect of CNF on nerve growth and regeneration.^3^ They showed that the CNF-based scaffold with the highest electrical activity exhibited the highest PC12 cell attachment and proliferation in the absence of cell adhesion molecules.^3^ They also reported increased osteoblast cell attachment and proliferation on the CNF-based nanocomposite with the highest electrical conductivity.^22^

Poly(glycolic acid) fibres incorporated with CNF showed osteoblast cell adhesion in an *in vitro* study, which was attributed to the high surface energy, surface chemistry and topography of the CNF incorporated in the polymer fibres.^24^ Furthermore, a CNF-based scaffold was studied for cardiac application.^25,26^ Martin *et al.* stated the improved conduction of electrical signals between cardiac cells with enhanced cell attachment and proliferation for a CNF-based polypropylene nanocomposite.^26^ The study by Mirzaei *et al.* showed that human endometrial stem cells could successfully differentiate into neuron-like cells on a CNF-based scaffold.^27^ A similar study was performed by Farzamfar *et al.*, which supported the successful nerve regeneration in a critical-sized sciatic nerve defect in a rat model.^28^ Prior studies reported the higher healing potential of CNF conductive scaffolds compared with non-conductive scaffolds.^25,29–31^ CNF are engineered as cylindrical nanostructures with a high aspect ratio, and excellent thermal conductivity, mechanical, and electrical properties, making them ideal for biological applications since cells require a three-dimensional matrix environment for efficient growth.^32^

In agreement with this, Sun *et al.* claimed that the presence of polypyrrole (Ppy) within a nerve guide would stimulate Schwann cell proliferation in early post-surgery and enhance myelin formation in later post-surgery.^33^ Similarly, Feng *et al.* reported the positive contribution of electric fields on the guided migration of neural stem cells, including their strong impact on functional recovery.^34^ A direct current electric field was highlighted as a cue to guide neurite growth and the migration of neurons and other types of cells.^34^ As reported by Stewart *et al.*, to achieve complete functional recovery after nerve injury, multiple cues are required during nerve regeneration, firstly modulating cell behaviour to guide regenerating axons to the distal nerve or eliciting migration then releasing growth factors to support cells.^35^ In the nervous system, neurons are the most important information messengers, which use electrical impulses and chemical signals called neurotransmitters for relaying information between the brain and the rest of the biological system. At the micro-level, neurons communicate with each other through neurotransmitters across a tiny space called the synapse between the axons and dendrites of adjacent neurons.

Herein, we aimed to develop a scaffold with printed lines to facilitate neuron migration to the printed lines and to support the growth of axons and dendrites towards a targeted end, which should lead to neuron communication *via* synapses. This new scaffold was developed by printing nanocomposite ink composed of carbon nanofibers (CNF) and DA on the surface of a polycaprolactone (PCL) film. One objective was to produce electrical conductivity to support the electrical signals produced by the nervous system to pass between the disconnected nerve and neurotrophic factors (NTTFs), which will assist in navigating the growth of neural cells in the required direction. These printed lines were composed of acid-functionalized CNF and an optimized concentration of DA in PCL, which formed a printable ink and prevented the rapid release of DA. Subsequently, the physical and biological properties of the scaffolds were evaluated.

## 2. Experimental

### 2.1. Materials and preparations

Poly(ε-caprolactone) (PCL, *M*_w_ = 57 000–90 000 g mol^−1^) was obtained from Sigma Aldrich, USA. Carbon nanofibers (CNF) (diameter: 60–150 nm and length: 30–100 μm) were purchased from Pyrograf Inc., USA. DA hydrochloride (*M*_w_ = 189.64 g mol^−1^) and *N*,*N*-dimethylformamide (DMF) (99.5%) (*M*_w_ 73.09 g mol^−1^) were obtained from Himedia, India. PCL film was prepared by dissolving 1 g of PCL pellets (10% w/v) in 10 mL DMF and heating it to 60 °C under magnetic stirring for 1 h. PCL films were formed using the solvent casting method in a Petri dish and dried for 72 h at room temperature. Carboxylated CNF (diameter 60–150 nm and length 30–100 μm, Pyrograf Inc., USA) were prepared following a previous study to enhance the CNF distribution in the polymer solution.^22^ In brief, 1 : 3 concentrated nitric acid (HNO_3_) and sulphuric acid (H_2_SO_4_) were used followed by washing with distilled water until the solution reached pH = 5.5.^22,36,37^ The nanocomposite ink was developed *via* the addition of 10% w/w functionalized CNF mixed in (10%) PCL solution, which was subjected to 20 min ultrasonication, followed by 2 h of magnetic stirring. In the final stage, different concentrations of DA were added to this nanocomposite ink and sonicated for 30 min. The details of the nanocomposite inks and their compositions are presented in Table 1.

**Table 1:** Details of the prepared materials

Ink code	Ink composition			Film composition
	Carboxylate CNF concentration (% w/w)	DA concentration (μg mL^−1^)	PCL (% w/w)	
P	0	0	10	PCL film (solvent casting from 10% w/w PCL in DMF solution); thickness = 300 ± 20 μm
PC	10	0		
PCD40	10	40		
PCD100	10	100		

### 2.2. Optimization of DA concentration

The concentration of DA was optimized based on the impact and toxicity of different concentrations of DA on the viability of neuro 2a cells. Neuro 2a cells, a mouse neuroblastoma cell line, were purchased from NCCS in Pune, India, and used for the cell viability analysis using the MTT assay.^22^ Different concentrations of DA (5–100 mg mL^−1^) were initially prepared in Dulbecco's Modified Eagle Medium (DMEM). In addition, concentrations in the range of 50–200 μg mL^−1^ were prepared in the PCL–CNF nanocomposite ink to identify the optimal concentration of DA. Additionally, 1 × 10^4^ cells were seeded on a 96-well plate for 24 h under a standard sterile cell culture environment. Then different treatments with DA were applied to the cells (*i.e.* different concentrations of DA prepared in culture medium and DA in ink). After the 48 h, MTT (thiazolyl blue tetrazolium bromide) was added to each of the 96-well plates and incubated for 4 h at 37 °C in a mammalian cell culture incubator. The medium containing MTT was then removed, and 200 μL dimethyl sulfoxide was added to each well to solubilize the formazan crystals. The absorbance of formazan was measured using a microplate reader at 570 nm, and the viability of the cells was calculated using the following equation:^38^

1 Cell viability (%) = absorbance of treated cells/absorbance of control cells × 100

The optimal concentration of DA was identified to be between 40 μg mL^−1^ and 100 μg mL^−1^ and used in the next section. Each concentration was tested by separating 9 wells in the same assay, and the results were averaged.

### 2.3. Printing nanocomposite ink

**Fig. 1:**
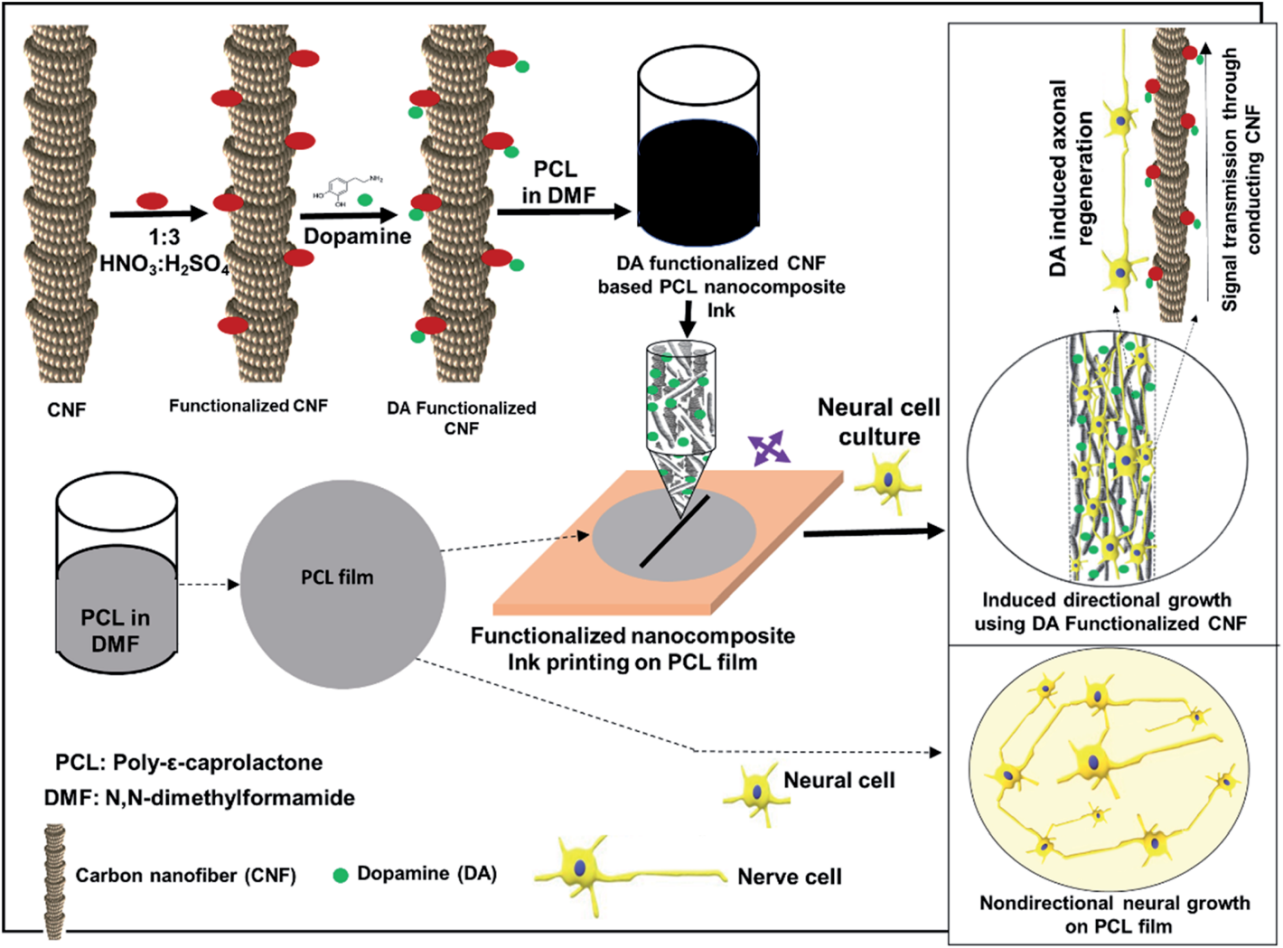
Schematic diagram of navigating directional growth using the functionalized nanocomposite ink.

CNF and CNF–DA aqueous suspensions (10%, 10%/40 μg mL^−1^ and 100 μg mL^−1^) were printed *via* an extrusion-based process using a bioprinter (Alfatek Systems, India), as shown in Fig. 1. A 5 mL syringe was fitted with a 20-gauge blunt-ended needle as the nozzle. The pressure in the syringe was adjusted to control the volumetric flow rate of ink through the nozzle. The CNF-based inks were printed in parallel lines with a distance of 5 mm between each line in a single layer on the prepared PCL film at room temperature (∼25 °C). The lines were deposited over a fixed time interval. The velocity of the print head was constant at 10 mm s^−1^. The codes of the printed materials with nanocomposite ink are P, PC, PCD40 and PCD100, and the details of the ink compositions are listed in Table 1. The materials were then dried for 24 h at room temperature.

#### Characterization of developed composite films

Microscope images of the P and PC films were captured using a Nikon DS Ri2 optical microscope at 4× magnification. The rheological properties of the inks were studied using a Modular Compact Rheometer (MCR 102), Anton Paar, Austria. The inks were tested in parallel plate mode with a 25 mm plate diameter, in which 1 mL of ink was placed between the plates and tested at a temperature of 37 °C, with a strain sweep in the range of 1 rad s^−1^ to 50 rad s^−1^. The tensile strength of these materials was tested using a Universal Testing Machine (UTM 3366), USA, with a 100 N load cell attached with pneumatic grips, set at a holding pressure of 4 bar. The specimen size for tensile testing was 40 mm × 5 mm × 0.3 mm (*L* × *W* × *T*), and the sample gauge was 20 mm. The dynamic mechanical properties of the materials were then tested using an MCR 102, with a frequency sweep in the range of 1 Hz to 10 Hz at 37 °C with 1% strain in universal extension mode (UXF), test fixture with a sample size of 40 × 5 × 0.3 mm (*L* × *W* × *T*) and gauge length of 30 mm. The impedance of the nanocomposite scaffolds was tested using an E4990A impedance analyser (Keysight Technologies, USA), equipped with a Kelvin clip test fixture (16089B). Before testing the materials, standard calibration steps were performed according to the manufacturer's instructions. The impedance values of the materials were recorded against frequency in the range of 20 Hz to 20 MHz. Five samples were tested for each experiment and the results were averaged.

### 2.4. Cell study

U87MG cells, which are human glioma cells and a representative of central neurons, were used to evaluate the biocompatibility and the possibility of supporting nerve growth on a selected path on each material. The materials were first cut into pieces of 1 cm × 1 cm and sterilized in 70% ethanol. Then, the materials were washed with DPBS for 15 min, followed by complete cell culture media (*i.e.* DMEM + 10% fetal bovine serum + 1% penicillin–streptomycin) for another 15 min. The U87MG cells (5 × 10^4^ cells per well of a 24-well plate) were then seeded on the materials and incubated overnight at 37 °C with 5% CO_2_. Then the incubated cells were gently transferred into another well-plate to examine cell attachment and proliferation. The attachment, migration, and proliferation of the cells were monitored using a ZOE™ Fluorescent Cell Imager (Bio-Rad Laboratories, USA).

The cytotoxicity of the materials was investigated using the MTT assay with U87MG cells. To perform the cell viability assay, the U87MG cells were seeded in a 24-well plate at a density of 5 × 10^4^ cells per well and incubated with the materials overnight at 37 °C in a 5% CO_2_ incubator. Then, the old medium was aspirated and complete medium added. Specifically, DMEM containing 0.5 g L^−1^ MTT (thiazolyl blue tetrazolium bromide) was added to each of the 24-well plates before incubating for another 4 h at 37 °C in an animal cell culture incubator. The old medium containing MTT was then removed, and 200 μL dimethyl sulfoxide (DMSO) was added to each well to solubilize the formazan crystals. The absorbance of formazan was monitored using a microplate reader (SpectraMax Paradigm Multi-Mode Microplate Reader, Molecular Devices, USA) at a wavelength of 570 nm with a 630 nm reference. The U87MG cells cultured with only complete DMEM were used as the control. The fraction of viable cells was calculated using eqn (1). Each composition was tested for the cell study 3 times and the results were averaged.

### 2.5. Scanning electron microscopy (SEM)

U87MG (human glioma) cells were seeded with a poly(l-lysine)-coated glass coverslip and the test materials at a density of 5 × 10^4^ cells per well and incubated at 37 °C in a 5% CO_2_ incubator. The cells were grown on the U87MG mixtures for 20 d before fixing them with 4% paraformaldehyde. The cells with a glass coverslip and PCL film were washed 3 times with PBS buffer. Then the cells were washed three times with 0.1 M sodium cacodylate buffer before fixing them with 1% osmium tetroxide for 30 min in the dark. After fixation, the cells were washed twice with deionized (DI) water. Finally, the cells were dehydrated with 50%, 70%, 95%, and 100% ethanol washes for 30 min. Following dehydration, the cells were critically point-dried and gold-coated with an SPI sputter coater.

### 2.6. Confocal laser scanning microscopy (CLSM)

U87MG (human glioma) cells seeded on the materials were cultured for 14 d according to the same protocol used for the cell attachment and proliferation assay.^38^ These materials were then fixed with 4% paraformaldehyde for 15 min, before washing with PBS buffer three times and incubated in 100% ethanol for 10 min. The materials were then washed twice using PBS, followed by staining of their cytoskeleton and cell nucleus. Diluted rhodamine-phalloidin and DAPI solutions were mixed at a ratio of 1 : 1 (v/v) to stain the cytoskeleton and the nuclei of the cells, respectively. The mixed dye solution was then added to each well containing the U87MG cells and incubated for 20 min. After incubation, the U87MG cell mixtures were washed twice using PBS buffer and observed and images captured under an N-STORM Super-Resolution confocal microscope.

### 2.7. Statistical analysis

Three independent experiments were performed and their average with standard deviation is plotted in the graphs. The Student's *t*-test was used to analyze the statistical significance between the developed PCL U87MG cell incubates using MS Excel.

## 3. Results and discussion

Several researchers have reported the potential concentration-dependent cytotoxicity of DA.^5,39^ A high level of DA in scaffolds can be toxic to cells and reduce cell attachment, differentiation and proliferation by causing cell death. Lan *et al.* investigated the effect of a high concentration of DA on human glioma cells and reported the anti-tumor (U87MG cells) activity of dopamine by inhibiting glioma cell proliferation.^20^ Thus, the dosage of DA in the nanocomposite ink was optimized by determining the cytotoxicity of the ink using cell viability testing, as shown in ESI Fig. S1(a).† This figure shows the viability of the neuro 2a cells with a DA concentration in the range of 5–100 μg mL^−1^. Different concentrations of DA were added to the nanocomposite ink, and the viability of the cells were reassessed after 48 h. The impacts of the different DA-based inks on the viability of the cells are shown in ESI Fig. S1(b).† They include a decrease in the viability of the cells with an increase in DA concentration in the medium to 40 μg mL^−1^, and 100 μg mL^−1^ resulted in the lowest cell viability. This indicates that DA concentrations above 100 μg mL^−1^ are cytotoxic, but no significant difference was found for concentrations in the range of 100 to 150 μg mL^−1^ in a similar study reported recently by Ganguly *et al.*^40^ They investigated the cytotoxicity of DA on cultured cells of neural origin to explore the mechanism of dopaminergic neurodegeneration in Parkinson's disease.^40^ Consistent with their studies and based on our results, 40 μg mL^−1^ and 100 μg mL^−1^ were selected as the optimal DA dosages, as shown in Fig. S2.†

**Fig. 2:**
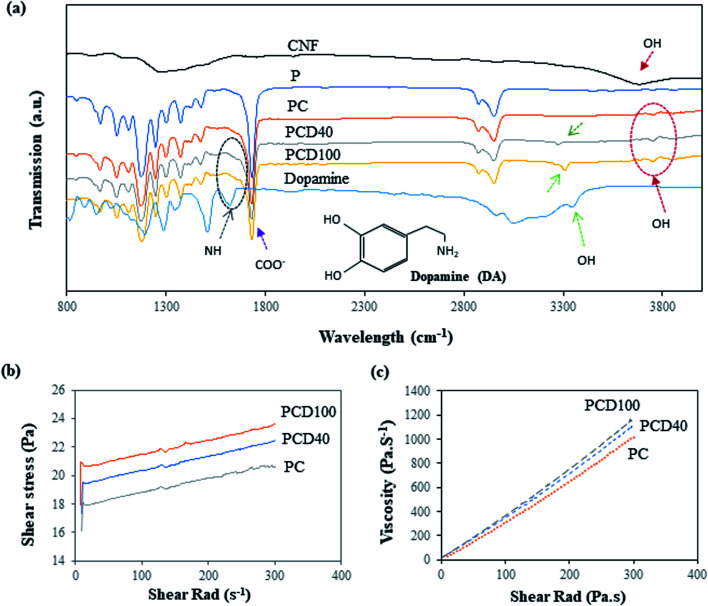
(a) FTIR spectra of pure PCL and PCL printed with CNF and DA (40 and 100 μg mL^−1^), where circles emphasize the OH peak (3700 cm^−1^) of the carboxylated CNF and NH peak (1565 cm^−1^) of dopamine. (b) Shear stress of the CNF and CNF + DA nanocomposite inks *versus* shear rate. (c) Viscosity *versus* shear rate of the prepared nanocomposite inks.

The Fourier transform (FTIR) infrared spectra of pure the PCL film and printed PCL film are shown in Fig. 2(a). The spectrum of the pure PCL film in Fig. 2(a) shows characteristic peaks at 1724 cm^−1^, 1367 cm^−1^, 1168 cm^−1^, and 2980 cm^−1^, corresponding to the vibrations of the C=O, CH, CH_2,_ and OH groups, respectively. In the printed PCL films, PCD40 and PCD100, the peaks of DA were observed at 3026 cm^−1^, 3265 cm^−1^, 3305 cm^−1^, and 1565 cm^−1^, which are assigned to stretching vibrations of the N–H, C–H, O–H, and C–H groups in DA, respectively. The peaks related to the carboxylated CNF are present at 1650 cm^−1^ and 3700 cm^−1^, corresponding to the C=O and OH groups, respectively. However, the peaks at 1565 cm^−1^ and 1650 cm^−1^ for DA and CNF, respectively, could not be clearly seen as they overlapped with the characteristic peaks of PCL. Thus, the peaks in the FTIR spectra confirm the existence of DA and CNF in the printed lines on the surface of the pure PCL film.

The rheological properties of the nanocomposite inks were investigated to assess their printability and the precision of the printed lines. Accordingly, shear stress and viscosity tests were performed, and the results are presented in Fig. 2(b) and (c), respectively. Fig. 2(b) shows that the shear rad increased on the addition of DA to the ink. This is due to the higher viscosity of the ink with DA in comparison with pure CNF, and the addition of extra DA resulted in an ink with higher viscosity. As is shown in Fig. 2(b and c), the developed ink is a shear thickening or dilatant fluid. This behaviour is often observed in suspensions and is due to the presence of CNF in the ink. The increasing shear rate led to alignment of the CNF in the fluid, where the CNF aligned against the plate, as shown in Fig. S1(b),† which means the ink behaved more like a solid than a solution. The viscosity of the PC, PCD40 and PCD100 nanocomposite inks at room temperature and 2 s^−1^ shear rad, was 35, 40 and 44 Pa s^−1^, respectively. Thus, high-pressure printing was necessary to provide better structural maintenance of the hierarchical line patterns in this study. Among the developed inks, PCD100 had the best printing quality with the sharpest contrast edges due to its high DA content, leading to a higher viscosity. Thus, the printed scaffolds with 0, 40 and 100 μg mL^−1^ DA concentration were selected and subjected to further physical characterization.

### 3.1. Physical characterization of the printed scaffold

**Fig. 3:**
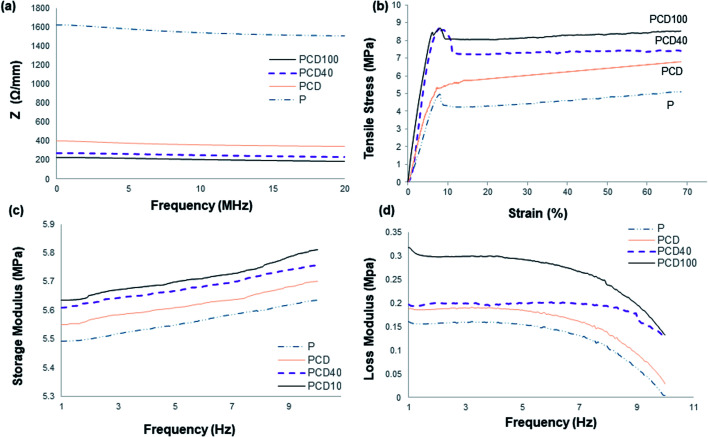
(a) Electrical resistance of pure PCL and printed PCL with CNF only and CNF with DA (40 and 100 μg mL^−1^) nanocomposite ink in the media. (b) Tensile strength of the developed materials. (c) Storage modulus and (d) loss modulus of pure PCL film and printed PCL film with CNF and DA (40 and 100 μg mL^−1^) nanocomposite ink.

The electrical resistance of the pure PCL film and printed lines on the PCL film in the media is shown in Fig. 3(a).

The pure PCL film was employed as the control. It had the highest electrical resistance and hence lowest electrical conductivity. Subsequently, the addition of CNF to the ink enhanced the electrical conductivity of the printed line on the PCL film, as confirmed by the high electrical conductivity of CNF. This improvement in electrical conductivity influenced the passage of electrical signals produced by the nervous system between the disconnected nerve ends, distal and proximal, which can enhance and direct the migration and proliferation of nerve cells. The positive effect of the electroconductive material and neurotrophic factors on enhancing the survival, maintenance and differentiation of neurons and axonal regeneration is well known in the literature.^5,6,12,14,15^ Thus, the addition of DA to the nanocomposite ink significantly enhanced its electrical conductivity, which was accompanied by an increase in the hydrophilicity of the ink and electrical conductivity of DA;^23^ however, the electrical conductivity did not change significantly with an increase in the concentration of DA in the ink. The presence of DA in the ink may reduce the resistance between the discontinuous CNF in the ink and lead to better ink conductivity. Furthermore, the signals and nerve impulses produced by the nerve cells/neurons can move across the synapse using chemicals called neurotransmitters, *e.g.* DA. Therefore, the addition of DA to the ink can assist and enhance the signal transmission between nerve cells (neurons).^41,42^ Zhang *et al.* and Kim *et al.* studied the effect of DA on the electrical properties of PPy.^43,44^ They found that the electrical properties of PPy were altered by the incorporation of DA in the polymer and the electrical conductivity of PPy increased to a maximum value, by almost two orders of magnitude, and then decreased with an increase in DA concentration.^43,44^ Su *et al.* reported a similar study with cellulose nano-paper and DA, and similar results were observed.^23^ This increase was ascribed to PDA being negatively charged with a negative zeta potential in water, and therefore could counterbalance the positive charge of PPY and act as a “dopant” to further improve the conductivity. Other studies showed the same trend after the addition of DA to CNF ink, where the electrical conductivity increased.^43,44^

Neural tissues are generally exposed to a combination of tensile, shear and compressive stresses in the *in vivo* environment during joint movement and muscle stretching, resulting in nerve excursion, strain and transverse contraction.^45^ Therefore, it is necessary to understand the mechanical behaviour of printed scaffolds. Several researchers have highlighted that an injured nerve can regenerate and elongate 1 mm d^−1^, which can affect the mechanical and structural properties of the nerve guides and tissue around the injured nerve.^45^ The average tensile property of a human cadaver median nerve has been reported to be about 2 N with a 7.5 mm extension.^45,46^ However, many factors influence the threshold of strain-induced nerve injury, where applying strain more than 11% is known to result in long-term damage to a common position in the upper limb. As reported in the literature, the blood flow is reduced by 50% with a strain of 11% and by as much as 100% with a strain of 15.7%. A prolonged reduction in blood flow alters nerve conduction and axonal support, which induces functional and histologic changes, including epineural and perineural tears, disrupted axons and significantly reduced nerve function. Consequently, treatment should be based on understanding the biomechanical properties of normal and pathological nerves. Therefore, in designing a nerve conduit, the biomechanical properties of the scaffold should be thoroughly investigated since they affect the success of the recovery and the final properties of the injured and recovered nerve. The biomechanical properties of the developed scaffolds were characterized *via* tensile (static) and dynamic mechanical analysis to determine the suitability of the developed scaffold for the treatment of nerve injuries. As reported in the literature, tensile strength is one of the important properties of nerve guides since nerve tissue experiences tensile stress during joint movement.^30,46^ The nerve elongates approximately 1 mm d^−1^ during repair, which affects the electro-physiochemical properties of the nerve tissue, for instance 0.5–1.5 mm d^−1^ femoral lengthening in the sciatic nerve of a rat reduced the signal transduction ability by 22–47%. Therefore, it is crucial to consider the tensile properties of the inner core when fabricating nerve guides.^31,46^

The tensile properties of the pure PCL film and printed PCL film with nanocomposite inks are illustrated in Fig. 3(b). As shown, the yield stress and strain of the developed scaffold are significantly higher than the normal peripheral nerve, resulting in effective elastic recovery after the application of high stress. High tensile strength is required since the scaffold will be used in *in vivo* media and it may undergo degradation, causing its mechanical strength to decrease significantly. This scaffold can be added to any area with nerve endings, *e.g.* knee joint junctions, and hence it should be able to withstand mechanical stress and shear. However, a very high tensile strength nerve guide with low strain can lead to a brittle scaffold and result in brittle fracture under a small bending strain, which can result from nerve damage by a sharp edge. Therefore, the mechanical properties of the scaffold should be optimal to support nerve growth, while accommodating bending (flexing) during application.

The yield strain and stress were higher for the printed films with the nanocomposite inks, which is due to the restriction applied by the ink to the movement of the polymer chains. Furthermore, this resulted from the large axial stiffness of CNF, amplifying the effect of compliance within the system when determining strain. This increase was higher for the nanocomposite ink with DA. This improvement is a result of the better adhesion of the ink to the surface of the PCL film due to the presence of the adhesive DA in the ink, which reduced the polymer chain motions and increased film rigidity under stress, leading to higher dislocation stress. The tensile stress at yield increased with the DA fraction. A higher DA concentration led to better interfacial adhesion between the PCL film and ink, and interfacial failure, as reveal by the cracked and separated lines from the film, occurred at lower stress for the ink without DA. This crack and interfacial failure began occurring at yield and continued until complete failure of the scaffold. The yield stress increased by 22%, 83% and 88% for PC, PCD40 and PCD 100 in comparison with P. The stiffness of the scaffold increased by 53%, 94% and 200% for PC, PCD40 and PCD100, respectively, in comparison with P. This is due to the better ink adhesion to the surface of the film, resulting in better stress transfer from the film to the ink, and restriction in polymer chain movement under the applied stress. It is important to note that the CNF ink lines could easily delaminate from the film, whereas at 40 μg mL^−1^ DA concentration, the bond between the ink and PCL film was so strong that the bond remained intact even under 8.6 MPa stress (12.5 N force). This significant improvement in adhesion is attributed to the presence of catechol functional groups on DA, which are known to cause good adhesion. Thus, the enhanced adhesion of the ink and mechanical properties of the PCD scaffold make it suitable for application as a nerve conduit.

The slope of the tensile stress *versus* strain curve in Fig. 3(b) gives the elastic modulus, which represents the stiffness of the nerve, approximately corresponding to *in situ* strain. As previously mentioned, PCD100 showed the highest stiffness of 187 MPa, followed by PCD40, PC and P with 59, 90 and 114 MPa, respectively. Thus, the results confirm that the presence of DA in the ink improved its attachment and adhesion the surface of the PCL films. This resulted in better stiffness and stress transfer between the PCL film and ink. The ink transferred the applied stress as a result of its good interfacial adhesion. Therefore, an increase in DA concentration led to better stress transfer, resulting in the highest tensile strength for PCD100.

Fig. 3(c and d) illustrate the viscoelastic properties of the materials, showing the same trend for dynamic mechanical property for all the developed scaffolds. As shown in Fig. 3(c), the storage modulus of the pure PCL film was found to be enhanced when printed with the nanocomposite ink. This resulted in the higher efficiency of elastic energy storage in comparison with the pure PCL film, which is due to the stiffness of the printed CNF ink. However, this improvement was higher for the scaffold with DA-CNF due to the adhesion of DA, resulting in better adhesion of the printed lines to the surface of the PCL film. This indicates that as the concentration of DA increased, the storage modulus significantly increased since the interfacial adhesion between the printed lines and film improved dramatically. Consequently, the stress transfer between the ink and film was enhanced upon the incorporation of the ink to carry the stress applied to the film. The highest storage modulus was obtained for PCD100, which was ∼5.84 MPa at a frequency of 10 Hz at 37 °C. The loss modulus (viscous property) of the printed PCL film, as shown in Fig. 3(d), demonstrated the high mechanical stability of the film due to the strengthening of the film by the printed lines to achieve improved mechanical properties. However, there was no significant difference between the inks with the two DA concentrations. Fig. 3(b and c) suggest that the printed lines restricted the movement of the films, leading to better mechanical properties and less flexibility. As expected, the final printed films with DA–CNF in the nanocomposite ink could hold their original shape for a reasonable time under stress application, which is required in the *in situ* environment. As the frequency increased, all the materials exhibited a reduction in their viscous property, indicating lower energy dissipation at higher frequencies. However, this frequency is not applicable for nerve application. PCD40 and PCD100 were the best candidates in terms of biomechanical properties (static tensile and dynamic mechanical properties) for nerve conduit application since the inks attached and adhered to the surface of the PCL film, resulting in enhanced interfacial properties between the ink and film, and both the ink and film carried the load and participate in the final mechanical properties of the scaffold.

### 3.2. *In vitro* cell culture studies

Effective nerve repair is necessary to bridge the gap between damaged nerve stumps. Accordingly, U87MG (human primary glioblastoma cells) cells were seeded on the pure and printed PCL films to investigate the effect of a printed line in supporting the path in a neural network. U87MG cells can migrate, proliferate, and differentiate quickly, while promoting the regeneration of injured peripheral nerves, as reported in the literature.^47,48^ Romao *et al.* investigated whether or not glial cells are the preferred substrate for neurons and have the same normal glial-neuron interactive properties with respect to neural growth and differentiation. They showed that human glioblastoma cells maintained the ability to support neuritogenesis.^49^ Furthermore, Zhao *et al.* showed that neural transcription factors induce the conversion of human glioma cells to neurons.^50^ Therefore, this cell line was a suitable choice for this study to evidently show the support of the scaffold for nerve growth.

Although all the components in the scaffolds, including PCL, CNF, and DA, are known to be biocompatible materials, it was necessary to understand the potential cytotoxicity levels of the scaffolds. Therefore, the MTT assay was performed to analyze the viability of the U87MG cells on both the pure PCL and printed PCL films. As shown in ESI Fig. S2,† the printed PCL scaffolds were non-toxic to the U87MG cells. Additionally, an increase in DA concentration provided more support for U87MG cell growth on the PCL matrix. As reported in the literature, CNF and DA support neural activity together with enhancing cell attachment, proliferation, and differentiation. Gopinathan *et al.* investigated the effect of CNF in a nerve conduit and reported that the scaffold with 10% CNF had the highest cell attachment and proliferation. Other studies also showed similar results.^3^

**Fig. 4:**
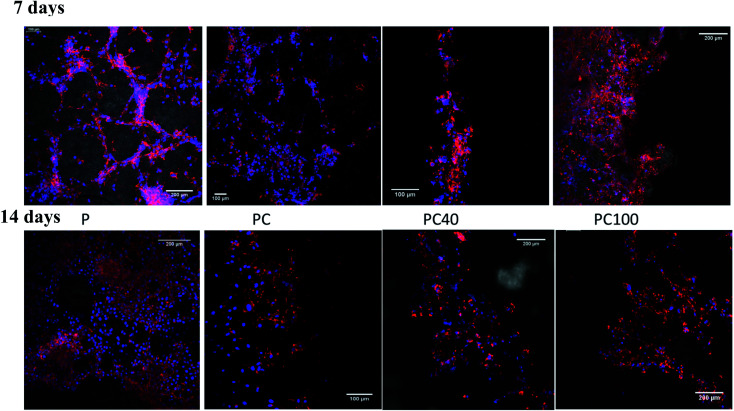
Confocal images of U87MG cells seeded on pure PCL and printed PCL after 7 and 14 d incubation.

**Fig. 5:**
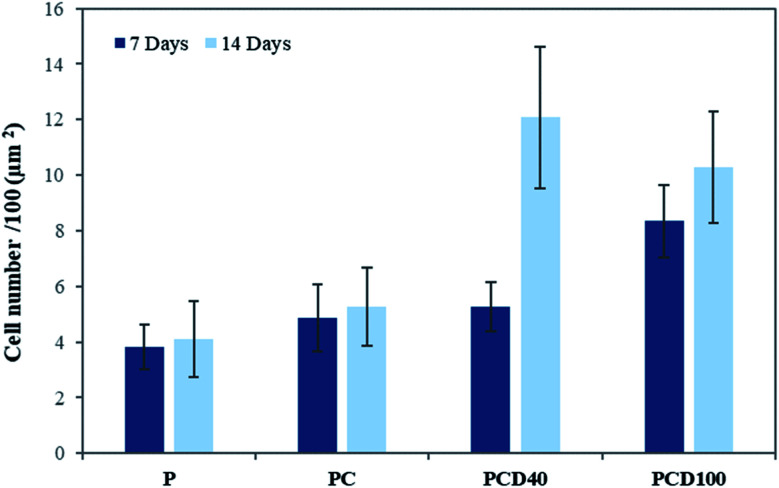
Number of cells on the scaffold after 7 and 14 d incubation.

The microstructure of printed lines combined with the microenvironment provided by CNF and DA had a positive impact on cell attachment and proliferation. Fig. 4 shows the cell attachment and proliferation in the printed area of the PCL films. It is evident from these confocal images that there is a higher number of cells on the printed lines compared with other areas of the PCL film. Furthermore, the cell count on the lines compared with that on the other areas confirmed this improvement, as shown in Fig. 5.

There was no significant change between P, PC and PCD40 after 7 days; however, PCD100 showed a significant increase in the number of attached cells to the scaffolds because of the release of DA to the environment in a shorter time than PCD40. This trend was different for 14 days of cell culture. However, the number for attached cells did not change significantly for P and PCD, PCD40, while PCD100 showed a dramatic improvement in the number of cells. This indicates that the nanocomposite ink, consisting of carboxylated CNF and DA, provided an appropriate microenvironment for neural cell attachment, proliferation, and axonal differentiation by stimulating the nerve tissues to send out new connections.

**Fig. 6:**
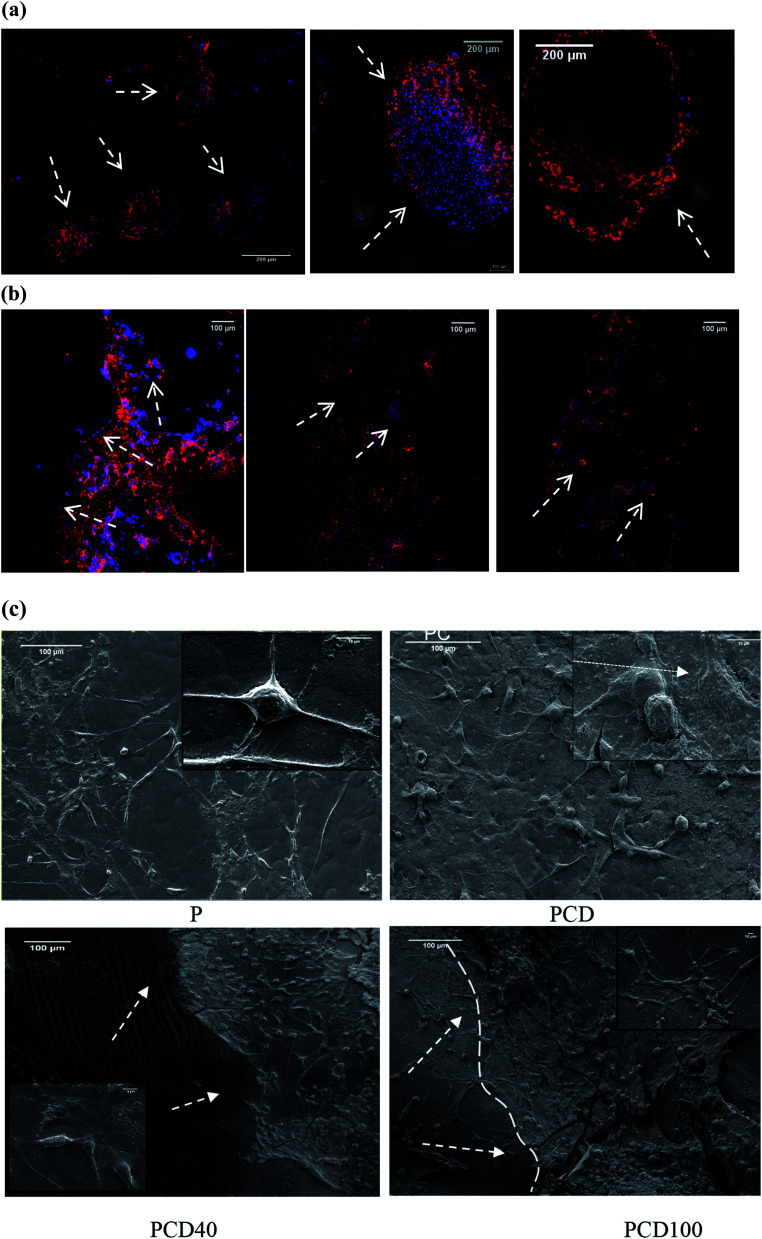
(a) Cellular cluster formed (indicated by arrows) on the pure PCL or pure PCL part of the printed film. (b) Fragmented parts of the printed ink after 14 d incubation (indicated by arrows). (c) SEM images of the pure PCL and printed PCL with ink seeded with U87MG cells after 20 d incubation (arrows and line show the interface between the ink and PCL film).

As shown in Fig. 6(a) and (b), cell proliferation on the PCL film appeared in the form of clusters (flower-like structure), while on the printed lines, neural cell growth was mostly observed along the printed lines, supporting the growth of the nerve cells along the selected path. Fig. 4(c) confirmed these results for the printed lines even without DA, although cell proliferation appeared to be more prominent in the DA-supplemented scaffolds.

The *in vitro* study of the PCL films revealed that the line patterns with a high concentration of DA disappeared faster compared with that with a lower DA concentration due to the rapid diffusion of DA to the media from the lines with a higher concentration of DA and degradation of PCL.^51^ This can explain the significant increase in cell attachment on PCD100 after 7 days in comparison with PCD40, indicating the burst release of DA to the environment after it was placed in the media due to the presence of a high concentration of DA on the PCL film.

As shown in Fig. 6(a–c), PCD40 still maintained the printed lines even after 14 days of incubation, unlike PCD100, which is due to its lower DA and higher PCL concentrations, and thus slower fragmentation.

Similarly, previous studies identified that scaffolds with a higher concentration of DA resulted in its faster release into the *in vitro* microenvironment.^4,16,51^ Therefore, the PCD40 scaffold appears to be the most favourable scaffold for possible nerve repair application due to the higher fraction of PCL in its ink and slower DA diffusion in the media, resulting in appropriate mechanical properties. Furthermore, the inclusion of functionalized CNF in the ink assists in maintaining the mechanical properties during the diffusion of DA and PCL degradation, providing more support for neural growth and neuron proliferation during the recovery time.

Thus, the *in vitro* results are consistent with the literature.^5,15,52,53^ Liu *et al.* demonstrated that DA enhanced the peripheral nerve regeneration *via* the promotion of the morphological and functional recovery in rat sciatic nerves.^15^ The regeneration of neurons by DA was examined by Berg *et al.*, confirming that DA provided the most extensive regenerative capacities in adult vertebrates.^18^ DA plays a vital role in nerve cell proliferation and repair *via* DA–DA receptor interaction, and the consequent activation of a signalling cascade.^12,22,41,42^ Thus, DA has been studied by many researchers for surface functionality modification and its high cell attachment ability.^14,36,37^ Similar results for cellular behaviour were observed in this study. It was identified that carboxylated CNF similarly possess potential regenerative properties, such as providing nano-topography, extracellular biomimetic nature, and conductive nature, which assist nerve cell and synapse communication with the target organ in addition to regrowth.

**Fig. 7:**
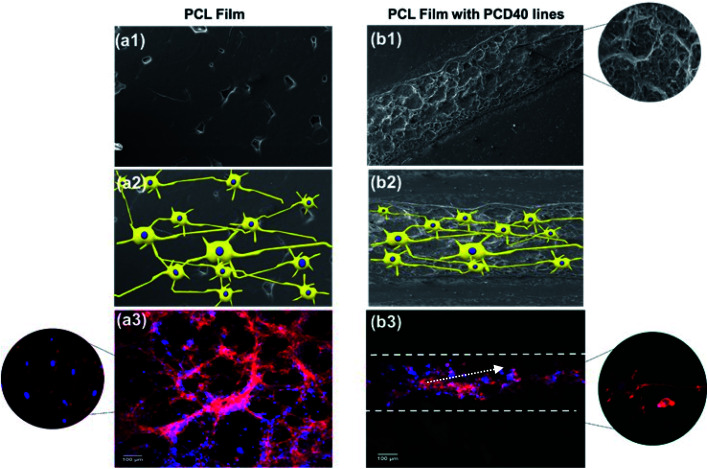
Growth and proliferation of neural cell networks on pure PCL film with no directional preference: (a1) SEM image, (a2) possible non-directional growth of nerves cells, and (a3) non-directional growth of glioma cells under fluorescence confocal microscopy (merged image – blue nucleus: DAPI and red actin: rhodamine phalloidin) and directional growth of neural cells on the CNF-based ink line drawn on the PCL film: (b1) SEM image of functionalized nanocomposite ink printed line on PCL film, (b2) possible directional growth of nerves cells, migration and movement to the printed lines then extending axons and dendrites, and (b3) induced directional growth of glioma cells under fluorescence confocal microscopy (merged image – blue nucleus: DAPI and red actin: rhodamine phalloidin); magnified images are shown as inserts.

The SEM images of the cell-seeded films (pure and printed PCL films) at the end of 20 days are shown in Fig. 4(d). The density of the nerve cells was found to be higher for PC, PCD40 and PCD100 in comparison with P. The SEM images show nerve cell attachment and axonal differentiation, especially on the printed lines with the highest amount of DA. The cells were observed to be in a healthy state with no obvious rounding and detachment. This confirms the positive contribution of DA on cellular proliferation and differentiation, which is useful for supporting the growth of neural cells along the required path. The SEM images of cell proliferation on the printed PCL scaffolds were consistent with the confocal images, as shown in Fig. 6(c). The red lines in Fig. 6(c) illustrate the borders of the printed ink on the PCL film. The neurons migrated and moved on the printed lines as their final location, and subsequently they extended axons and dendrites, allowing them to communicate with other neurons *via* synapses, leading to the establishment of functional neural circuits, which mediate sensory and motor processing.^54,55^ This movement and migration play an essential role in the final sensory and motor function. In this investigation, the printed lines provide rich microenvironments, which navigate this movement and migration, thus directing the axonal growth and dendrites, as shown in Fig. 7(b1).

As illustrated in Fig. 7, consistent with the microscopy images, a high density of neural growth was observed on the printed lines containing the functionalized nanocomposite ink. This is because the ink provides an excellent microenvironment for neuron movement and migration, as shown in Fig. 7(b1), in comparison with the pure PCL film due to the presence of DA and CNF. Therefore, for the film with printed lines, the neurons migrated to these areas and then extended into axons and dendrites, whereas for the pure PCL film, this migration did not occur, and extending axons and neuron occurred randomly as a flower-shaped growth.

Thus, DA in the nanocomposite stimulated the nerve tissues to send out new connections, while CNF repaired the broken neural circuits. Accordingly, it can be concluded that the scaffold with the printed ink, composed of DA and CNF, is a suitable choice as a nerve conduit material because it provides a supporting environment for the growth of neurons and axons. The combined effects of CNF and DA provide a suitable microstructure and microenvironment to support neural cells in damaged areas to grow along suitable pathways to reconnect at the targeted distal end.

## 4. Conclusion

The benefit of a new scaffold design and materials, DA and CNF printed lines, is to support neuron migration and axonal growth toward the targeted end of an injured nerve by providing an optimal pathway. There is potential for this newly developed scaffold to promote a connecting nerve growth route by providing a suitable microenvironment for neuron migration, while stimulating nerve tissue to extend new connections *via* DA and repair broken neural circuits *via* CNF. The extension of axons and dendrites in an optimal direction after neuron migration leads to effective communication with other neurons *via* synapses, resulting in the establishment of neural circuits, which mediate sensory and motor processing.

The presence of functionalized CNF in the conductive ink to imprint scaffolds led to the controlled release of DA, which provided support for neural growth and neuron proliferation during the recovery time. The printed PCL films showed thermomechanical properties comparable with human nerves. This new printed scaffold with nanocomposite ink shows potential for guiding and supporting neural cell migration, growth and extension of axons and dendrites. This is due to the composition of the conductive printed lines, which provided high affinity to neural cells, resulting in improved neural cell movement, migration, attachment, and growth on the printed lines. Therefore, this newly developed scaffold will be an excellent candidate for nerve engineering applications.

## Conflicts of interest

The authors declare no competing financial interest.

## Supplementary Material

RA-010-D0RA06556K-s001
