# Correction: Three-dimensional directional nerve guide conduits fabricated by dopamine-functionalized conductive carbon nanofibre-based nanocomposite ink printing

**DOI:** 10.1039/d0ra90129f

**Published:** 2020-12-10

**Authors:** Shadi Houshyar, Mamatha M. Pillai, Tanushree Saha, G. Sathish-Kumar, Chaitali Dekiwadia, Satya Ranjan Sarker, R. Sivasubramanian, Robert A. Shanks, Amitava Bhattacharyya

**Affiliations:** School of Engineering, College of Science, Engineering and Health, RMIT University Melbourne 3001 Australia shadi.houshyar@rmit.edu.au; Tissue Engineering Laboratory, PSG Institute of Advanced Studies Coimbatore-641004 India; Functional, Innovative and Smart Textiles, PSG Institute of Advanced Studies Coimbatore-641004 India abh@psgias.ac.in; RMIT Microscopy and Microanalysis Facility, College of Science, Engineering and Health, RMIT University Melbourne 3001 Australia; Department of Biotechnology and Genetic Engineering, Jahangirnagar University Savar Dhaka-1342 Bangladesh; Electrochemistry Laboratory, PSG Institute of Advanced Studies Coimbatore-641004 India; School of Science, College of Science, Engineering and Health, RMIT University Melbourne 3000 Australia

## Abstract

Correction for ’Three-dimensional directional nerve guide conduits fabricated by dopamine-functionalized conductive carbon nanofibre-based nanocomposite ink printing’ by Shadi Houshyar *et al.*, *RSC Adv.*, 2020, **10**, 40351–40364, DOI: 10.1039/D0RA06556K.

The authors regret that an incorrect version of Fig. 2 was included in the original article. The correct version of Fig. 2 is presented below.

**Fig. 2:**
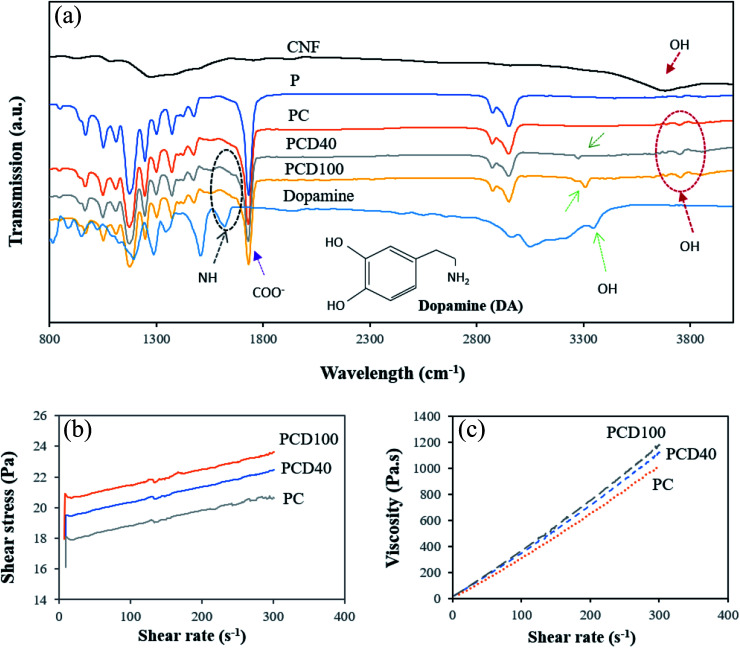
(a) FTIR spectra of pure PCL and PCL printed with CNF and DA (40 and 100 μg mL^−1^), where circles emphasize the OH peak (3700 cm^−1^) of the carboxylated CNF and NH peak (1565 cm^−1^) of dopamine. (b) Shear stress of the CNF and CNF + DA nanocomposite inks *versus* shear rate. (c) Viscosity *versus* shear rate of the prepared nanocomposite inks.

The Royal Society of Chemistry apologises for these errors and any consequent inconvenience to authors and readers.

## Supplementary Material

