# Supplementary material for: Three-dimensional directional nerve guide conduits fabricated by dopamine-functionalized conductive carbon nanofibre-based nanocomposite ink printing
Source: RSC Adv. 2020 Nov 9;10(66):40351–64. doi: 10.1039/d0ra06556k (PMC9057509; doi:10.1039/d0ra06556k)
Supplement: RA-010-D0RA06556K-s001 [file RA-010-D0RA06556K-s001.pdf]

Electronic Supplementary Material (ESI) for RSC Advances.
This journal is © The Royal Society of Chemistry 2020

Supplementary Figures

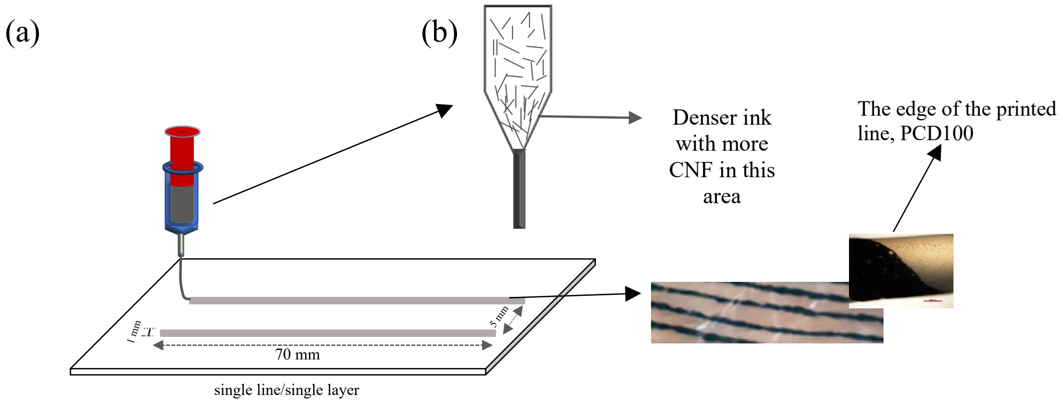

Fig. S1. Schematic of the (a) printing paths (b) printing nozzle

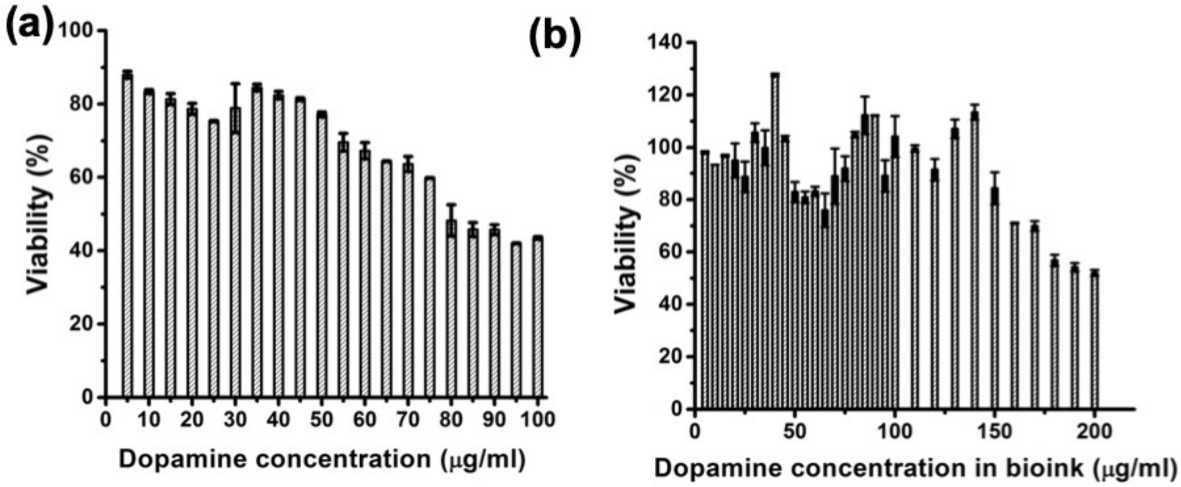

Fig. S2. Dopamine concertation versus cell viability after 48h; (a) pure dopamine, (b) dopamine in biomaterial ink, biomaterial ink consists of various dopamine concertation with 10% CNF (concentration of dopamine in μg/ml)

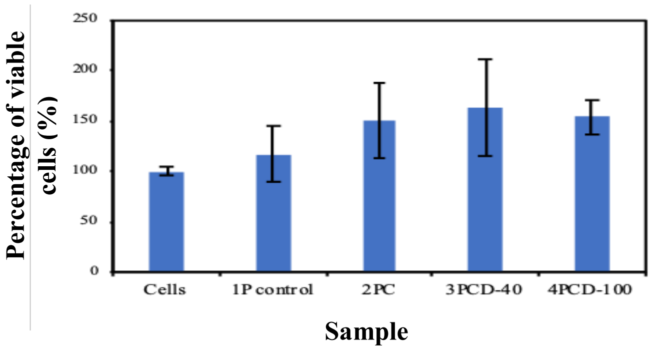

Fig. S3: MTT assay for developed samples (PCL film printed with biomaterial ink (consist of 40 µg/ml and 100 µg/ml dopamine and 10 %CNF in PCL) using U87MG cells (human glioma cells)
